# A device for ventilation-analogue mechanostimulation *in vitro*

**DOI:** 10.1186/cc10714

**Published:** 2012-03-20

**Authors:** K Gamerdinger, M Schneider, E Smudde, J Guttmann, S Schumann

**Affiliations:** 1 University Medical Center Freiburg, Germany

## Introduction

The mechanical stress-strain characteristics of most living tissues is nonlinear and frequency dependent. During spontaneous breathing the mechanical strain on the pulmonary tissue is akin to a sinusoidal profile. In contrast, during mechanical ventilation the stimulation profile of the lung tissue differs considerably from a sinusoidal pattern. While all *in vitro *experiments aiming at dynamic stimulation typically use sinusoidal patterns, we here describe the establishment of a new device affording a ventilation-analogue stimulation pattern, allowing a better imitation of the situation *in vivo*. The new device includes a linear motor connected to four piston pumps and it allows the identical stimulation of four probes at the same time. Here we show how we stressed four test samples with sinusoidal, rectangular and ventilation-analogue mechanostimulation and how we analyzed them for frequency contents by means of a fast-Fourier transform.

## Methods

Silicone membranes serving as substitutes for biological tissue samples were placed inside a bioreactor [1] either in single-membrane or in double-membrane configuration. Cyclic mechanostimulation at repetition rates ranging from 15/minute to 2,000/minute at amplitude volumes of 0.5 up to 2.8 ml, corresponding to a surface increase of 5% up to 100%, were used. The system was driven with sinusoidal, rectangular and ventilation-analogue profiles simulating the ventilatory pattern which is associated with the volume-controlled ventilation.

## Results

The drive system allowed us to vary the amplitude from 0 up to 100% surface increase. At amplitudes of 0.5 and 1.0 ml we were able to apply a frequency range from 0 up to 2,000/minute, and at an amplitude of 2.0 ml a frequency range from 0 up to 800 sinusoidal deflections per minute. We were able to apply the rectangular and the ventilation-analogue volume patterns to the probes. Close inspection of the pressure curves revealed that rapid volume increases were followed by peaks with subsequent relaxation decays when rectangular or ventilation-analogue stimulation patterns were applied. The frequency spectra of the pressure variation revealed side frequencies of up to 10 Hz for the rectangular mechanostimulation profile.

## Conclusion

With our new mechanostimulation system we are able to configure the frequency content of the applied strain profile and furthermore to identify the frequency content of the resulting stress on the tissue.
